# Blockade of Tyrosine Kinase, LCK Leads to Reduction in Airway Inflammation through Regulation of Pulmonary Th2/Treg Balance and Oxidative Stress in Cockroach Extract-Induced Mouse Model of Allergic Asthma

**DOI:** 10.3390/metabo12090793

**Published:** 2022-08-25

**Authors:** Saleh A. Alqarni, Abdulwahab Bineid, Sheikh F. Ahmad, Naif O. Al-Harbi, Faleh Alqahtani, Khalid E. Ibrahim, Nemat Ali, Ahmed Nadeem

**Affiliations:** 1 Department of Pharmacology and Toxicology, College of Pharmacy, King Saud University, Riyadh 11451, Saudi Arabia; 2 Department of Zoology, College of Science, King Saud University, Riyadh 11451, Saudi Arabia

**Keywords:** allergic asthma, LCK, Th2 cells, Treg cells, eosinophils

## Abstract

Asthma is one of the most common inflammatory diseases affecting the airways. Approximately 300 million individuals suffer from asthma around the world. Allergic immune responses in the asthmatic airways are predominantly driven by Th2 cells and eosinophils. Lymphocyte-specific protein tyrosine kinase (LCK) is a non-receptor tyrosine kinase which regulates several key intracellular events through phosphorylation of its substrates. Some of the intracellular signaling pathways activated by LCK phosphorylation help in differentiation of Th2 cells which secrete allergic cytokines that amplify airway inflammation. Therefore, this investigative study was designed to determine the role of LCK in a cockroach extract (CE)-induced airway inflammation murine model of allergic asthma. Further, the effect of a pharmacological LCK inhibitor, A-770041, on allergic airway inflammation and key intracellular pathways in CD4+ T cells was assessed. Our data exhibit that there is an activation of LCK during allergic airway inflammation as depicted by increased *p*-LCK levels in CD4+ T cells. Activated LCK is involved in the activation of ITK, PLC-γ, GATA3, NF*k*B, and NFATc1. Activated LCK is also involved in the upregulation of Th2 related cytokines, such as IL-4/IL-5/IL-13 and oxidative stress, and the downregulation of Treg cells. Furthermore, utilization of LCK inhibitor causes the reduction in *p*-LCK, PLC-γ, GATA3, and NFATc1 as well as Th2 cytokines and oxidative stress. LCK inhibitor causes upregulation of Treg cells in allergic mice. LCK inhibitor also caused a reduction in CE-induced airway inflammation and mucus secretion. Therefore, the inhibition of LCK signaling could be a fruitful approach to adjust allergic airway inflammation through the attuning of Th2/Treg immune responses. This study could lead to the design of newer treatment options for better management of allergic inflammation in asthma.

## 1. Introduction

One of the most common inflammatory diseases affecting the airways is asthma. The symptoms of asthma include wheezing, shortness of breath, and coughing [1]. There are multiple risk factors that can promote the pathogenesis of asthma such as smoking, air pollution, indoor allergens, and occupational chemicals [2]. Asthma has world-wide prevalence, affecting approximately 300 million individuals around the world. In the United States, there were around 25 million people who had asthma in 2010. Estimates in 2013 showed that asthma put a huge economic burden on health care system costing around $80 billion [3,4,5]. In Saudi Arabia also, asthma is classified as one of the most common chronic diseases [6]. 

The pathophysiology of asthma is complex since it involves a variety of cells (originating from innate and adaptive immune arms) such as lymphocytes, eosinophils, macrophages, dendritic cells, and neutrophils. Apart from immune cells, non-immune cells such as epithelial cells serve a crucial role in the recruitment of innate/adaptive immune cells thus amplifying allergic airway inflammation [1,2]. The inflammation of the airways develops due to the release of a large number of inflammatory mediators, such as cytokines, chemokines, and oxidants expressed/produced by different immune and non-immune cells [7]. These inflammatory mediators act in concert to cause characteristic features of allergic asthma such as eosinophilic infiltration into the airways and mucus hypersecretion [2,8].

One of the most-common non-receptor tyrosine kinases that acts as mediators of signal transduction is the SRC kinase family. Lymphocyte-specific protein tyrosine kinase (LCK) belongs to the non-receptor SRC family and regulates a number of cellular processes, such as cell multiplication and polarization [9,10]. LCK is found intracellularly and executes an essential role in T cells function through regulation of T-cell receptor (TCR) signaling. When TCR on naïve CD4+ T cells (Th0) cells is activated by an antigen-presenting cell, this leads to the phosphorylation and activation of LCK [10,11]. Active LCK plays a crucial role in phosphorylation of tyrosine residues of different proteins such as ZAP-70, ITK and PLCγ in Th0 cells which are required for activation of downstream protein transcription factors such as NFATc1, NF*k*B, and GATA3 in Th0 cells [12,13,14]. Activation of these downstream signaling molecules promotes the expression/release of proinflammatory allergic cytokines, e.g., IL-4, IL-5, and IL-13 in Th2 cells [13]. These transcription factors may be involved in transcriptional regulation of oxidative enzymes such as iNOS and NADPH oxidase which may participate directly or indirectly in regulation of Th2 immune responses and oxidative inflammation [15,16].

Multiple reports have shown that Th2 cells are responsible for the promotion of airway inflammation as they cause recruitment of eosinophils into the airways, which is a characteristic feature of allergic asthma [2,17]. The GATA-3 transcription factor is responsible for development of Th2 cells which perpetuate airway inflammation and mucus hypersecretion by constantly releasing IL-5, IL-4 and IL-13 during chronic allergen exposure [18]. Furthermore, Th2 cytokines are also chemoattractant for eosinophils [17]. NFATc1 is also reportedly involved in regulation of Th2 cells along with GATA3 through modulation of Th0 differentiation into Th2 cell [19,20]. Thus, both could affect the expression of allergic cytokines in Th2 cells, however their relationship with LCK in the context of allergic airway inflammation has not been investigated yet.

Therefore, this study examined the role of LCK and its relationship with downstream signaling pathways in CD4+ T cells in a cockroach extract (CE)-induced airway inflammation model of allergic asthma in mice. Further, LCK inhibitor was utilized to assess its potential in curbing allergic airway inflammation. Our data display that LCK gets activated by exposure to CE allergens and is associated with airway inflammation and mucus hypersecretion. Inhibition of LCK signaling leads to significant reduction in eosinophilic infiltration and mucus hypersecretion along with attenuation of Th2 related cytokines such as IL-4/IL-5 in CE-exposed mice.

## 2. Materials and Methods

### 2.1. Animals

The current study employed C57BL/6 mice (9–10 weeks of age) for the investigation of different parameters in allergic mouse model. These mice were received from the Experimental Animal Care Center, College of Pharmacy, King Saud University. The mice were kept in specific pathogen-free conditions under established protocol to provide normal housing environment (24–26 °C ambient temperature with a light/dark cycle of 12 each). Mice were provided with continuous supply of food and water at will. The use of animals in this study was approved by the Animal Care and Research Committee of the College of Pharmacy, King Saud University and all protocols for conducting different experiments were followed as per the recommendations of the committee. 

### 2.2. Development of Cockroach Extract (CE)-Induced Allergic Inflammation in Mice

Mice were sensitized for 5 successive days with cockroach extract (CE), 50 uL intranasally (i.n.) per mouse once daily followed by rest period of 5 days for development of adaptive immune responses. Ten days after first i.n. sensitization, mice challenged with 50 uL CE i.n. for 5 successive days [21]. On day 16, mice were sacrificed by deep inhalational anesthesia using isoflurane followed by various analyses (BAL procedure/histopathology, and molecular/biochemical experiments).

### 2.3. Experimental Groups

To explore whether blockade of LCK signaling has any modulation on CE-induced allergic responses, mice were i.n. (50 uL/mouse) administered with small molecule pharmacological LCK inhibitior, A-770041 (dissolved in dimethylsulfoxide; Medchem Express, Monmouth Junction, NJ, USA) at a dose of 10 mg/kg for 5 consecutive days, 30 min before CE challenge (day 11–15). Experimental mice were randomly selected and categorized into the following groups: Group A: Control group (CON): mice were administered saline i.n. during sensitization and challenge phase and drug vehicle in the challenge phase. Group B: Control group administered LCK inhibitor (CON+LCK-inhibitor): mice were administered saline i.n. during sensitization and challenge phase and LCK inhibitor at 10 mg/kg, (i.n.) in the challenge phase as written above. Group C: CE-administered group (CE): mice were administered CE i.n. during sensitization and challenge phase as written above. Group D: CE group administered LCK inhibitor (CE+LCK-inhibitor): mice were administered CE i.n. during sensitization and challenge phase and LCK inhibitor at 10 mg/kg, (i.n.) in the challenge phase as written above.

### 2.4. BAL Procedure

BAL was conducted on day 16 according to the standard criteria as explained earlier [22]. Briefly, trachea of each mouse was cannulated after terminal anesthesia followed by washing of airspaces with cold PBS (pH-7.4). Recovered BAL fluid was centrifuged to get the pellet of leukocytes, which was suspended in 1 mL cold PBS (pH-7.4) followed by counting in hemocytometer to get total leukocyte count. Slides for differential leukocyte count were prepared using cytocentrifuge followed by Diff-Quik staining. BAL cells on the Diff-Quik stained slides were counted under bright field microscopy as per the standard morphological criteria to obtain the differential leukocyte count. Results are expressed as cells/mL BAL.

### 2.5. Immunofluorescent Analysis of LCK Signaling and Different Th2 Related Markers in Pulmonary Cell Suspension Using Flow Cytometry

Lungs were isolated and chopped into very small pieces using fine surgical scissors followed by incubation in digestion cocktail containing collagenase and DNAse I in RPMI-1640. This led to formation of single cell suspension of pulmonary cells which was then utilized for flow experiment as done earlier [22]. Single cells from pulmonary tissue were first incubated with monoclonal antibodies against cell surface antigen, i.e., CD4 (BioLegend, San Diego, CA, USA) fluorescently conjugated to APC-Cy7/APC/FITC. After the normal step of fixation and permeabilization (Miltenyi Biotech, Bergisch Gladbach, North Rhine-Westphalia, Germany) of pulmonary cells in the suspension, cells were then labeled with monoclonal antibodies against intracellular proteins, such as *p*-LCK, *p*-ITK, *p*-PLCγ, NFATc1, *p*-NFkB, IL-4, IL-5, IL-10, Foxp3, GATA-3, iNOS, and Nitrotyrosine (R&D Systems, Minneapolis, MN, USA; BioLegend, San Diego, CA, USA). Immunolabeled leukocytes in pulmonary cell suspension were then run and 10,000 events were acquired on a flow cytometer (Beckman Coulter, Indianapolis, IN, USA), followed by evaluation of fluorescently conjugated intracellular/cell surface proteins using Cytomics FC 500 software as stated before [22]. 

### 2.6. Evaluation of Allergic Cytokine in the Lung by ELISA

Assessment of pulmonary IL-4 protein levels was conducted using commercial ELISA kit (R&D Systems, Minneapolis, MN, USA) according to the protocol of the supplier. Data are presented as pg/mg protein. 

### 2.7. Evaluation of mRNA Expression by Real-Time PCR

Pulmonary tissue was dissected in RNAlater solution at the end of the study and frozen for a month, after which total RNA was isolated using Trizol method. Thereafter, 0.5μg RNA was transformed into cDNA using High-Capacity reverse transcription cDNA archive kit (Applied Biosystems, Grand Island, NY, USA) according to the protocol of the manufacturer as described previously [22]. Expression levels of mRNA expression of IL-4, IL-13, GATA3, NFATc1, MUC5AC, GAPDH (using primers from GenScript, Piscataway, NJ, USA) was evaluated by real-time PCR on ABI PRISM 7500 sequence detection system (Applied Biosystems, Grand Island, NY, USA) as stated before [22]. Relative levels of mRNA expression among different groups were evaluated by well-established delta delta Ct method [23].

### 2.8. Histopathology of the Lung Tissue Using H&E and PAS Staining

Lungs were dissected at the end of the experiment and fixed in 10% phosphate-buffered formalin. Lung tissue was sectioned at 5 μm thickness using a microtome followed by staining with hematoxylin and eosin (H&E) stain (for inflammation related morphology) and PAS stain (for mucus production). Slides after staining were fixed and observed under a bright field microscope. 

### 2.9. Chemicals and Reagents

Chemicals/reagents in this investigation were of the highest quality and were procured from Sigma Chemicals (St. Louis, MO, USA) if not stated otherwise. 

### 2.10. Statistical Analysis

The data are presented as mean± SEM. Biochemical/molecular parameters in different groups were analyzed using one-way ANOVA (analysis of variance) followed by Tukey’s multiple comparison tests. Results were considered statistically significant if *p* < 0.05.

## 3. Results

### 3.1. LCK Is Activated during Allergic Airway Inflammation and Its Inhibition Causes Reduction in Downstream Pathways in CD4+ T Cells of CE-Exposed Mice

**Figure 1:**
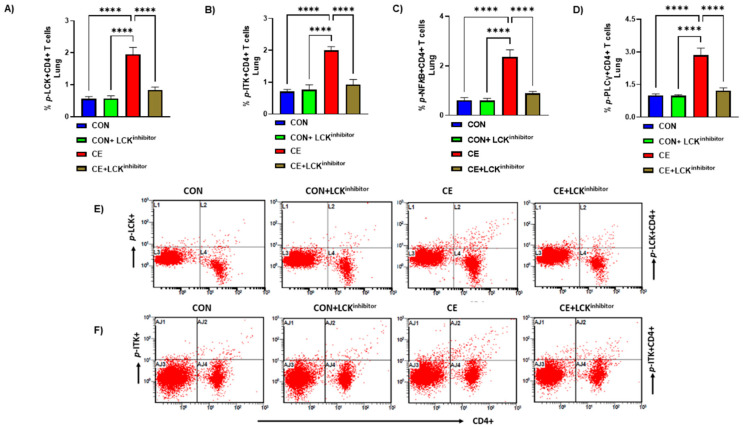
Activation of LCK signaling and its mitigation by LCK inhibitor in CD4+ T cells of CE-exposed allergic mice. (**A**) % *p*-LCK+CD4+ T cells, (**B**) % *p*-ITK+CD4+ T cells, (**C**) *p*-NF*k*B+CD4+ T cells, (**D**) *p*-PLCγ+CD4+ T cells, (**E**) Flow plot with representative depiction of double positive immunostaining in CD4+ T cells, i.e., *p*-LCK+ immunoreactivity in CD4+ T cells, and (**F**) Flow plot with representative depiction of double positive immunostaining in CD4+ T cells, i.e., *p*-ITK+ immunoreactivity in CD4+ T cells. Data are presented as mean ± SE, *n* = 6/group. **** *p* < 0.0001.

LCK is a crucial tyrosine kinase with respect to T cell signaling. However, its function with respect to CE-induced allergic airway inflammation remains unexplored. Therefore, it was first assessed whether there is any modulation of LCK signaling during CE-induced allergic airway inflammation. Our data show that LCK signaling is activated in CD4+ T cells during allergic inflammation, as depicted by elevated % of phosphorylated LCK (*p*-LCK) immunostaining in CD4+ T cells (Figure 1A,E). Next, we explored whether elevated *p*-LCK+CD4+ T cells were associated with the modification of intracellular signaling pathways downstream of LCK such as ITK, PLCγ, NFkB. Our data show that there is increased % of *p*-ITK immunostaining in CD4+ T cells in CE-exposed mice (Figure 1B,F). % of *p*-PLCγ and *p*-NFkB immunostaining in CD4+ T cells was also elevated in CE-exposed mice (Figure 1C,D). Furthermore, we wanted to confirm whether LCK signaling in CD4+ T cells was indeed responsible for activation of these intracellular pathways. For this purpose, we utilized small molecule LCK inhibitor A-770041 to delineate the role LCK in allergic inflammation. Inhibition of LCK signaling by A-770041 led to a reduction in the percentage of *p*-LCK+CD4+ T cells as well as *p*-ITK+CD4+ T cells, *p*-PLCγ+, and *p*-NFkB+ CD4+ T cells (Figure 1A–F). These data reveal that LCK is activated in CD4+ T cells in CE-exposed mice and responsible for the activation of key downstream pathways.

### 3.2. Inhibition of LCK Signaling Causes Reduction in Airway Inflammation in CE-Induced Allergic Model of Asthma

**Figure 2:**
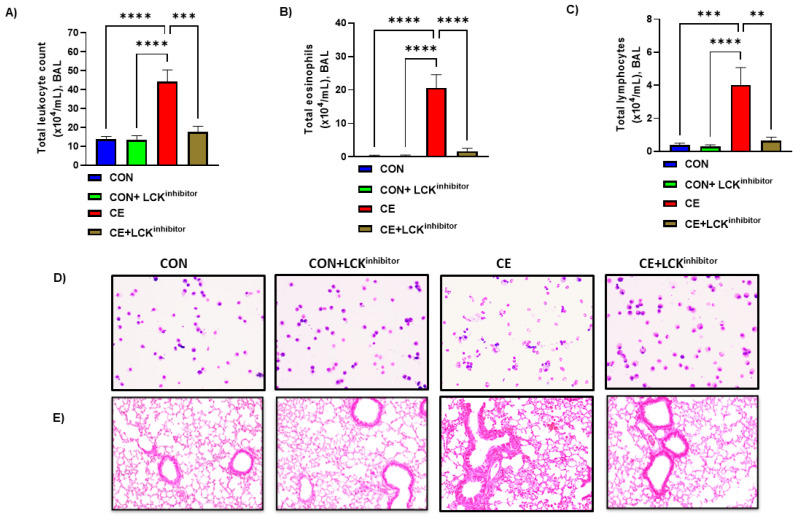
Amelioration of allergic airway inflammation by LCK inhibitor in CE-exposed allergic mice. (**A**) Total leukocytes count in BAL, (**B**) Total eosinophils in BAL, (**C**) Total lymphocytes in BAL, (**D**) Diff-Quik staining of BAL cells, and (**E**) H&E staining of lung tissue. Data are presented as mean ± SE, *n* = 6–8/group. ** *p* < 0.01; *** *p* < 0.001; **** *p* < 0.0001.

Next, we investigated whether LCK signaling was involved in CE-induced allergic airway inflammation in mice. For this purpose, LCK inhibitor A-770041 was administered i.n. before each CE exposure for 5 successive days followed by evaluation of airway inflammation and mucus hypersecretion. Our data display that total leukocyte count, eosinophilic count and lymphocytic count were elevated in CE exposed mice (Figure 2A–D). Further, H&E staining showed marked infiltration of leukocytes in alveolar spaces in CE-exposed mice confirming the establishment of allergic airway inflammation (Figure 2E). Blockade of LCK signaling by A-770041 led to significant reduction in allergic airway inflammation as displayed by total leukocyte count, eosinophilic count, and lymphocytic count in BAL (Figure 2A–D). H&E staining also confirmed BAL cellularity data as LCK inhibitor A-770041 caused a marked reduction in leucocytic infiltration around airway spaces in CE-exposed mice (Figure 2E).

**Figure 3:**
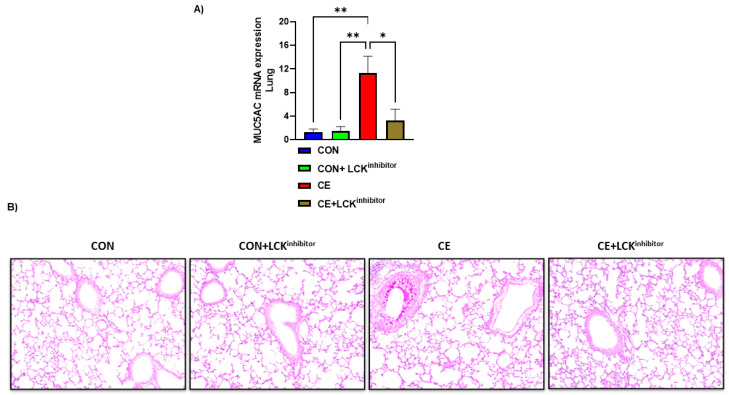
Reduction in mucus production by LCK inhibitor in CE-exposed allergic mice. (**A**) Muc5Ac mRNA expression in lung, (**B**) PAS staining of lung tissue. Data are presented as mean ± SE, *n* = 6/group. * *p* < 0.05; ** *p* < 0.01.

Further, mucus production was assessed by PAS staining and expression of MUC5AC in the lung of CE-exposed mice and those mice treated with A-770041. Our data show that CE-exposed mice have significant upregulation in pulmonary MUC5AC expression and PAS staining in airway cells (Figure 3A,B). Blockade of LCK signaling by A-770041 led to a significant reduction in pulmonary MUC5AC expression and PAS staining in airway cells in CE-exposed mice (Figure 3A,B). These data show that the blockade of LCK signaling has the capability to attenuate CE-induced airway inflammation and mucus hypersecretion.

### 3.3. Blockade of LCK Signaling Leads to Attenuation of Th2 Related Transcription Factor Signaling in CE-Exposed Mice

**Figure 4:**
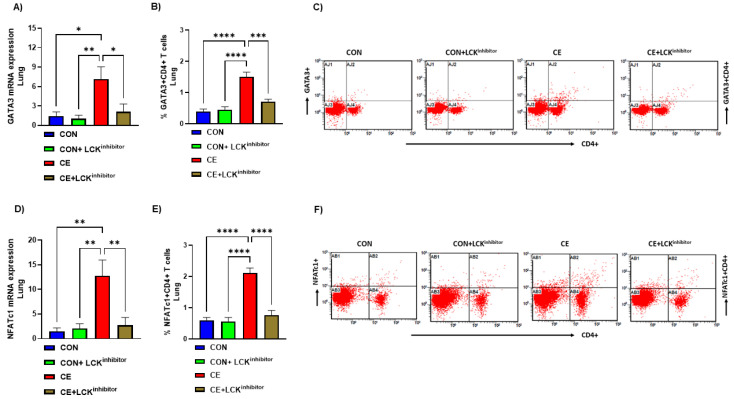
Attenuation of Th2 transcription factor signaling by LCK inhibitor in CE-exposed allergic mice. (**A**) GATA3 mRNA expression in lung tissue, (**B**) % GATA3+CD4+ T cells, (**C**) Flow plot with representative depiction of double positive immunostaining in CD4+ T cells, i.e., GATA3+ immunoreactivity in CD4+ T cells, (**D**) NFATc1 mRNA expression in lung tissue, (**E**) % NFATc1+CD4+ T cells, and (**F**) Flow plot with representative depiction of double positive immunostaining in CD4+ T cells, i.e., NFATc1+ immunoreactivity in CD4+ T cells. Data are presented as mean ± SE, *n* = 6/group. * *p* < 0.05; ** *p* < 0.01; *** *p* < 0.001; **** *p* < 0.0001.

Past investigations reveal that LCK may be an essential kinase in regulation of Th2 cell homeostasis, however whether it affects CE-mediated modulation of transcription factors remains unexplored. Therefore, effect of CE exposure was assessed on transcription factors such as GATA3 and NFATc1 which are essential for Th2 differentiation. Our data reveal that pulmonary GATA3 mRNA expression and % of GATA3+CD4+ T cells were increased CE-exposed mice (Figure 4A–C). Further, pulmonary NFATc1 mRNA expression and % of NFATc1+CD4+ T cells were also increased in CE-exposed mice (Figure 4D–F). Next, it was explored whether the blockade of the LCK pathway modulates these transcription factors in CD4+ T cells in CE-exposed mice. Our results display that the blockade of LCK signaling by A-770041 led to marked mitigation in the expression of GATA3 and NFATc1 in CD4+ T cells of CE-exposed mice (Figure 4A–F). This was demonstrated by a reduction of double positive immunostaining of GATA3 as well as NFATc1 in CD4+ T cells and pulmonary tissue in CE-exposed mice (Figure 4A–F). These data display that blockade of LCK signaling leads to a reduction in Th2 related immune responses in allergic mice.

### 3.4. Blockade of LCK Signaling Leads to Attenuation of Th2 Related Cytokines in CE-Exposed Mice

**Figure 5:**
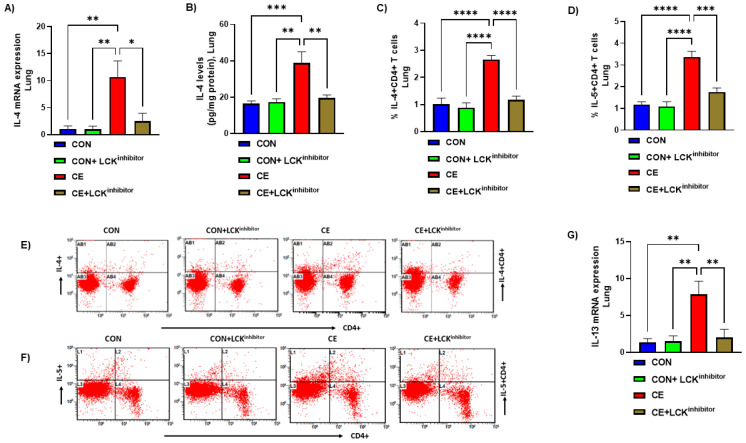
(**A**) IL-4 mRNA expression in lung tissue, (**B**) IL-4 protein levels in the lung tissue, (**C**) % IL-4+CD4+ T cells, (**D**) % IL-5+CD4+ T cells, (**E**) Flow plot with representative depiction of double positive immunostaining in CD4+ T cells, i.e., IL-4+ immunoreactivity in CD4+ T cells, (**F**) Flow plot with representative depiction of double positive immunostaining in CD4+ T cells, i.e., IL-5+ immunoreactivity in CD4+ T cells, and (**G**) IL-13 mRNA expression in lung tissue. Data are presented as mean ± SE, *n* = 6/group. * *p* < 0.05; ** *p* < 0.01; *** *p* < 0.001; **** *p* < 0.0001.

Next, we examined if LCK signaling is also required for expression of inflammatory cytokines involved in allergic airway inflammation. Our results show that levels of Th2 cytokines in pulmonary CD4+ T cells and tissue were markedly elevated in CE-exposed mice as displayed by elevated levels of IL-4 mRNA expression, IL-4+CD4+T cells and IL-4 protein levels (Figure 5A–C,E). Further, another important Th2 cytokine, IL-5 was also elevated in CD4+ T cells as depicted by increased % of IL-5+CD4+ T cells (Figure 5D,F). mRNA expression of another important Th2 cytokine, IL-13 was also found to be elevated in CE-exposed mice (Figure 5G). Next, it was explored if blockade of LCK pathway modulates these Th2 related cytokines in CE-exposed mice. Our results display that blockade of LCK signaling by A-770041 led to marked mitigation in the expression of IL-4/IL-5/IL-13 in pulmonary compartment of CE-exposed mice (Figure 5A–G). It was displayed by a reduction of IL-4/IL-13 mRNA expression. IL-4 protein levels, % of IL-4+CD4+ cells, and % of IL-5+CD4+ T cells in CE-exposed mice (Figure 5A–G). These data display that blockade of LCK signaling leads to a reduction in Th2 related allergic cytokines in CE-exposed mice.

### 3.5. Blockade of LCK Signaling Leads to Augmentation of Treg Cells in CE-Exposed Mice

**Figure 6:**
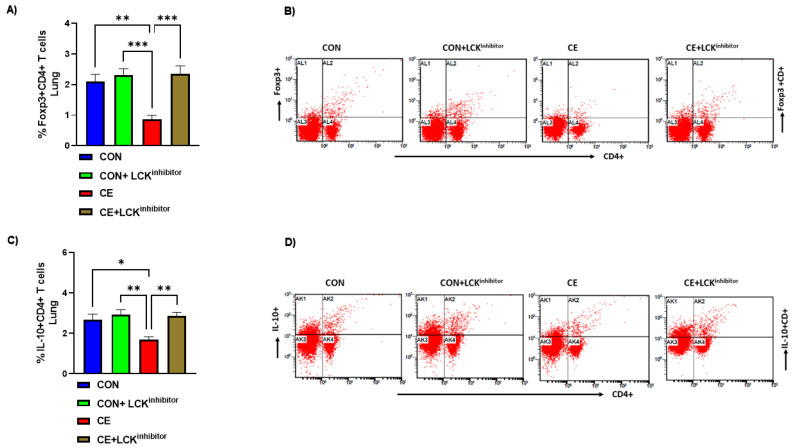
Augmentation of Treg cells by LCK inhibitor in CE-exposed allergic mice. (**A**) % Foxp3+CD4+ T cells, (**B**) Flow plot with representative depiction of double positive immunostaining in CD4+ T cells, i.e., Foxp3+ immunoreactivity in CD4+ T cells, (**C**) % IL-10+CD4+ T cells, and (**D**) Flow plot with representative depiction of double positive immunostaining in CD4+ T cells, i.e., IL-10+ immunoreactivity in CD4+ T cells. Data are presented as mean ± SE, *n* = 6/group. * *p* < 0.05; ** *p* < 0.01; *** *p* < 0.001.

It was also investigated whether LCK signaling blockade affected any modulation of Treg cells in CE-exposed allergic mice. Our data reveal that mice with allergic airway inflammation presented with reduction in Treg immune responses in the pulmonary compartment. This was depicted by decreased % of Foxp3+CD4+ T cells and IL-10+CD4+ T cells in CE-exposed allergic mice (Figure 6A–D). When CE-exposed were treated with LCK inhibitor, there was a marked elevation in Treg immune responses in the lung. This was evident from increased % of Foxp3+CD4+ T cells and IL-10+CD4+ T cells in CE-exposed allergic mice (Figure 6A–D). These data show the augmentation of Treg cells by LCK inhibitor which may be partly responsible for the amelioration of airway inflammation in allergic mice.

### 3.6. Blockade of LCK Signaling Leads to Attenuation Oxidative Stress in CD4+ T Cells in CE-Exposed Mice

**Figure 7:**
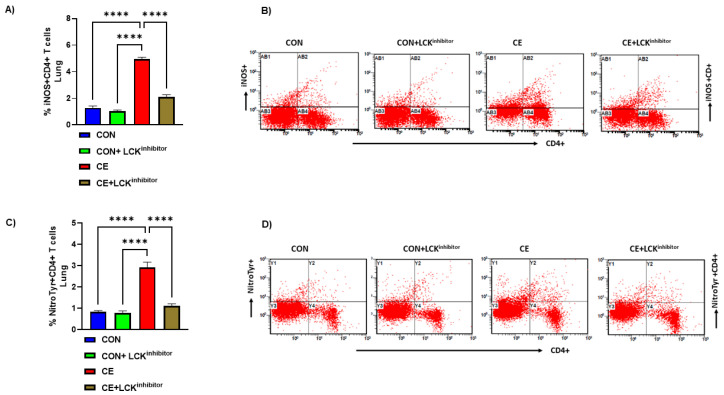
Attenuation of iNOS and nitrotyrosine in CD4+ T cells by LCK inhibitor in CE-exposed allergic mice. (**A**) % iNOS+CD4+ T cells, (**B**) Flow plot with representative depiction of double positive immunostaining in CD4+ T cells, i.e., iNOS+ immunoreactivity in CD4+ T cells, (**C**) % NitroTyr+CD4+ T cells, and (**D**) Flow plot with representative depiction of double positive immunostaining in CD4+ T cells, i.e., NitroTyr+ immunoreactivity in CD4+ T cells. Data are presented as mean ± SE, *n* = 6/group. **** *p* < 0.0001.

Lastly, it was interrogated if blockade of LCK signaling had any effect on oxidative stress in CE-exposed allergic mice. Our data show that allergic mice had increased % of iNOS+CD4+ T cells and Nitrotyr+CD4+ T cells in CE-exposed allergic mice (Figure 7A–D). Administration of allergic mice with LCK inhibitor led to mitigation of oxidative stress as depicted by reduced % of iNOS+CD4+ T cells and Nitrotyr+CD4+ T cells (Figure 7A–D). Overall, these data show the mitigation of Th2 cells and oxidative stress, as well as the augmentation of Treg cells by LCK inhibitor, which in combination may cause a reduction in airway inflammation in CE-exposed allergic mice.

## 4. Discussion

**Figure 8:**
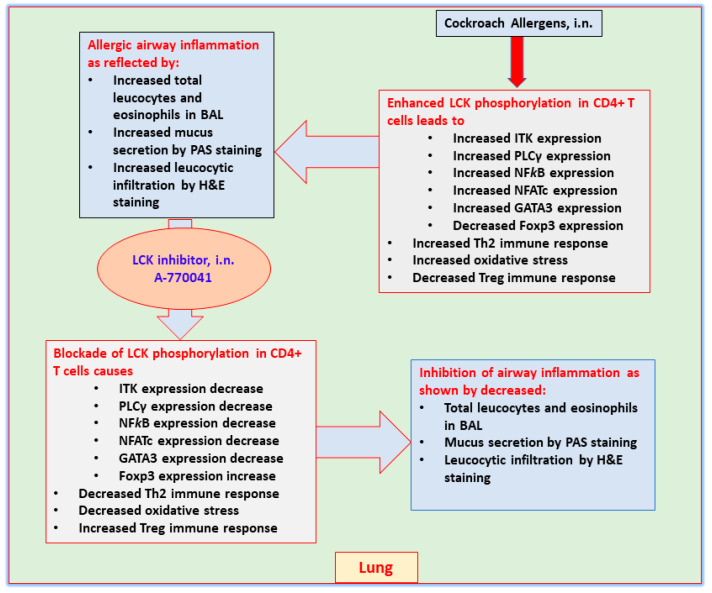
Presumptive mechanism of action of LCK inhibitor, A-770041 on airway inflammation in CE-induced mouse model of asthma based on the observations of this study.

Asthma is a chronic airway disorder presenting in heterogenous phenotypes. Allergic asthma constitutes one of the most predominant phenotypes and is characterized by presence of Th2 immune cells and infiltration of eosinophils into airspaces, thus displaying the close interaction between innate and adaptive immune cells. Activation of toll like receptors (TLRs), such as TLR4 and protease activated receptor-2, by components present in common household allergens, such as cockroach extract allergens and house dust mite, cause the expression/production of complex inflammatory mediators from airway epithelial cells and dendritic cells [17,24,25]. These mediators activate T cells to produce allergic cytokines, such as IL-4, IL-5, and IL-13, that initiate and amplify airway inflammation and mucus production [3]. The current investigation displays that CE-exposed mice have increased *p*-LCK levels in CD4+T cells which is linked with allergic airway inflammation and Th2 related immune responses. This was confirmed by LCK inhibitor that led to mitigation in CE-induced airway inflammation and mucus production as well as allergic cytokines, IL-4/IL-5/IL-13 (Figure 8).

Tyrosine kinases constitute a significant proportion of total kinases found in human genome and are implicated in various downstream signaling events which are coupled with surface receptors on immune and non-immune cells [26,27] Due to their established involvement in the pathogenesis of various autoimmune/inflammatory diseases, tyrosine kinases have stood out as remarkable therapeutic targets in various diseases. One such tyrosine kinase is LCK which has been reported to be an important participant in the pharmacological control of vast array of diseases like cancer and immune-mediated autoimmune and inflammatory conditions [10,28,29,30]. LCK may play an important role inflammatory pulmonary diseases such as asthma as it is well-known that LCK controls expression and release of various allergic cytokines through differentiation/polarization of Th2 cell subtype. However, its role in an asthma model relevant to human asthma remained unexplored until now.

There are multiple allergic airway inflammation models in animals that take advantage of using common allergens prevalent in our indoor or outdoor environment such as house dust mite, cockroach allergens and ragweed [31]. The animal models relating to asthma contribute to the discovery of new potential treatments of asthma. In addition, the animal models of asthma facilitate our interpretation regarding the pathophysiology of airway inflammation. CE allergens are common indoor allergens and are ubiquitously present in our homes. Further, CE allergens are commonly associated with allergic airway inflammation through continuous daily exposure to susceptible subjects [31]. Allergic airway inflammation in the CE-exposed is predominantly driven by Th2 cells and eosinophils [24,25]. Therefore, this model was utilized in the current investigation to evaluate the role of LCK in allergic asthma.

Activation of CD4+ T cells is driven by engagement of TCR with MHCII on antigen presenting cells such as dendritic cells. This leads to activation of LCK and its downstream signaling in CD4+ T cells. In the absence of TCR engagement, CD3/TCR complex is unphosphorylated, however TCR ligation initiates phosphorylation of several proximal signaling molecules such as ITK and PLCγ [11,32,33]. Our study also showed increased *p*-LCK, which led to increased *p*-ITK and *p*- PLCγ in CD4+ T cells, indicating that activation of these pathways was dependent on LCK activation as evident by utilization of LCK inhibitor. Restriction of these proximal signaling by LCK inhibitor could be responsible for attenuation of Th2 related immune responses in CE-exposed mice.

Th2 cell differentiation is dependent on several transcription factors such as GATA3 and NFATc1 [34]. Our study showed increased GATA3 and NFATc1 expression in CD4+ T cells in CE-exposed mice which was downregulated markedly by LCK inhibitor. This observation suggests that LCK signaling blockade might reduce Th2 immune responses through downregulation in both of these transcription factors. Earlier studies have shown decreased production of allergic cytokine IL-4 in CD4+ T cells isolated from LCK deficient mice which was due to aberrant expression of GATA3 [12]. Impaired Th2 immune responses have also reported in ITK deficient CD4+ T cells. ITK deficiency in CD4+ T cells caused a reduction in allergic cytokine IL-4 due to impaired translocation of NFATc [13]. Since our study showed decreased allergic airway inflammation by LCK inhibitor, it is likely that the reduction in Th2 immune responses in CE-exposed mice by LCK inhibitor is due to a combined effect on downstream signaling molecules, such as ITK, NFATc1, and GATA3, in this study.

Th2 cytokines, such as IL-4 and IL-5, have been reported to play a crucial function in asthmatic airways due to their potential in augmentation of allergic inflammation. These cytokines are chemotactic for eosinophils which are key contributors in causing allergic airway inflammation [1,35,36]. Our study showed a reduction in CE-induced eosinophilic infiltration into the airspaces by LCK inhibitor, which may be due to mitigation of LCK-mediated signaling in CD4+ T cells. Mucus production was also downregulated by LCK inhibitor which could probably be due to reduction in IL-4/IL-5/IL-13 as Th2 cytokines are known to augment allergen-induced mucus production in the lung [35,37]. Therefore, the blockade of LCK signaling is capable of restricting key features of asthmatic airways, i.e., eosinophilic inflammation and mucus hypersecretion.

Previous investigations have revealed a strong involvement of Treg cells in allergic airway inflammation. Treg cells are important in the context of allergen-induced eosinophilic inflammation as Treg cells have the potential to suppress Th2 immune responses which are cardinal for the development of allergic airway inflammation [38,39,40]. Our study also showed the downregulation of Treg cells with concurrent augmentation of Th2 immune responses in pulmonary compartment of CE-exposed allergic mice. Th2 related cytokines and transcription factors have been reported to cause suppression of Treg immune responses [38,41,42]. Upregulation of Treg cells in this study could be a consequence of LCK inhibitor-mediated mitigation of Th2 immune response in CE-exposed allergic mice.

T cells have also shown to be responsible for production of oxidative stress through expression of oxidative enzymes [15,16,43]. Our study showed upregulation of oxidative stress in CD4+ T cells in mice with allergic asthma as depicted by upregulated iNOS and nitrotyrsoine in CD4+ T cells. Treatment with LCK inhibtior led to the inhibition of iNOS and consequent reduction in nitrotyosine in CD4+ T cells. LCK signaling is known to regulate NF*k*B in T cells which is a master transcription factor for regulation of inflammatory genes such as iNOS [15,44]. Therefore, it is likely that LCK-mediated downregulation of iNOS is via inhibition of NF*k*B expression in CD4+ T cells. Earlier studies have shown that oxidative enzymes, such as iNOS and NADPH oxidase, may be responsible for upregulation of Th2 immune responses [16,45,46,47]. Overall, LCK-mediated reduction in oxidative stress could be partially responsible for the attenuation of allergic airway inflammation in CE-treated mice.

This study is an in vivo study where it is difficult to delineate exact proximal and distal molecular pathways related to LCK signaling. In this study, we have shown probable proximal substrates, e.g., ITK and PLCγ, and distal substrates, e.g., NF*k*B, GATA3, NFATc of LCK signaling in CD4+ T cells of allergic and LCK-treated allergic mice. However, to ascertain the precedence of one pathway over the other in LCK signaling cascade would require specific inhibitors of each pathway and an in vitro approach using isolated CD4+ T cells and antigen presenting cells (e.g., dendritic cells). This is part of our future work and a limitation of this study.

Inhibitors of LCK have been reported to be efficacious in T cell-driven inflammatory diseases that include psoriasis, multiple sclerosis, lupus, and arthritis [14,27,48,49,50]. The LCK inhibition approach was tested in an ovalbumin asthma model in mice earlier [29]. However, this study did not delineate whole spectra of LCK signaling in CD4+ T cells and its downstream effects on Treg cells and oxidative stress. Further, it was tested systemically using an siRNA approach unlike the intranasal delivery of the drug to the lung in our study. Intranasal delivery of small molecule pharmacological inhibitor is more relevant to human asthma and likely to mitigate side effects produced by the global inhibition of LCK using systemic therapy. Further, our study emphasizes the role of LCK signaling in an allergic model relevant to human asthma, unlike ovalbumin which is not a natural allergen. Finally, our study suggests that the LCK inhibition strategy may have therapeutic potential in the treatment of allergic airway inflammation.

## Data Availability

The authors confirm that all data underlying the findings are fully available without restriction. All relevant data are within the paper.
